# Bacterial diversity changes in response to an altitudinal gradient in arid and semi-arid regions and their effects on crops growth

**DOI:** 10.3389/fmicb.2022.984925

**Published:** 2022-10-14

**Authors:** Maryam Zakavi, Hossein Askari, Mohammad Shahrooei

**Affiliations:** ^1^Department of Plant Sciences and Biotechnology, Faculty of Life Sciences and Biotechnology, Shahid Beheshti University, Tehran, Iran; ^2^Department of Microbiology and Immunology, Clinical and Diagnostic Immunology, KU Leuven, Leuven, Belgium

**Keywords:** soil, plant–microbe interaction, elevation gradient, arid, altitudes, bacteria

## Abstract

The microbiome of soil has a fundamental role in maintaining the health of soil and plants. While the diversity of microbes is one of the most important factors in the environment, little is known about the effects of elevation on the microbiome and the impact of the affected microbiome on plants. The main goal of this study is to expand our knowledge of what happens to the soil bacterial community along an altitudinal gradient and investigate their possibly different impacts on plant growth. Bacteria from soils at various altitudes have been isolated, characterized, and identified by Matrix-Assisted Laser Desorption/Ionization-Time of Flight Mass Spectrometry (MALDI-TOF MS) to determine the effects of an elevational gradient on the microbiome and plant growth. Furthermore, their effects have been investigated by isolates assessment on maize, wheat, and canola. Based on our results, higher altitude results in a higher diversity of the microbiome and lower bacteria biomass. *Bacillus cereus* is found in abundance in arid and semi-arid samples. Interestingly, enhanced diversity in higher altitudes shows similarity in response to environmental stress and tolerates these factors well. Furthermore, the inoculation of these bacteria could enhance the overall growth of plants. We prove that bacterial communities could change their biomass and diversity in response to altitude changes. These indicate evolutionary pressure as these bacteria could tolerate stress factors well and have a better relationship with plants.

## Introduction

Climate globally mediates the biodiversity-ecosystem stability relationship (García-Palacios et al., 2018). The extreme conditions of the desert climate continuously negatively impact the reduction of species diversity (Wall and Virginia, 1999). The soil microbiome is essential for maintaining soil fertility, cycling nutrients, and carbon sequestration (Jacoby et al., 2017; Jin et al., 2022). Additionally, the soil microbiome affects the health of plants and animals in a variety of environments, both directly and indirectly (Bender et al., 2016). It is also responsible for micro and macro nutrition metabolism and catabolism (Islam et al., 2020). Soil microbial diversity indicates a significant relationship with multifunctionality drivers such as climate, soil factors, and spatial predictors (Delgado-Baquerizo et al., 2016) and is dynamically influenced by water content, temperature, and biological activities (Allen et al., 2011; Wilpiszeski et al., 2019). To date, a variety of studies have investigated the response of the microbial community in bulk soil to elevational gradients, suggesting that elevational gradients strongly affect the microbial diversity and community composition of bulk soils by altering plant and soil characteristics (Meng et al., 2013; Li et al., 2018; Saitta et al., 2018; Guo et al., 2020). Based on bacterial taxonomic diversity, contradicting results have been found in temperate regions, with taxonomic richness varying along elevational gradients; either decreasing or increasing (Bryant et al., 2008), showing a hump-backed relationship (Singh et al., 2012) or not showing any relationship (Fierer et al., 2011; Shen et al., 2014; Zhang et al., 2015; Merino-Martín et al., 2020). Elevational gradients have been used as proxies for the impacts of climate change on above and belowground organisms, including plants and soil microbes (Yao et al., 2017; Donhauser and Frey, 2018; Adamczyk et al., 2019). Additionally, it is documented that investigating the change in bacterial communities along the altitudinal gradient may shed light on the prediction of future climate change scenarios (Yuan et al., 2014). Since it is known that individual microbial taxa exhibit different life strategies, determining the effects of elevational gradients on the relative abundances of specific microbial taxa may be important for interpreting patterns of microbial diversity and understanding ecosystem functioning (Yao et al., 2017). Thus, elevational gradients provide unique natural laboratories to study the response of soil bacteria to the environmental drivers of arid and semi-arid regions under various light conditions, temperature, humidity, height, and other possible factors. Microbial populations are key components of the soil–plant system, where they are placed in a network of interactions that affect plant development (Lugo et al., 2008). The soil microbiome governs the biogeochemical cycling of elements (Jansson and Hofmockel, 2018), decomposition (Ren et al., 2016; Fierer, 2017), metabolizing of external contaminants (Wang et al., 2018), soil carbon sequestration, and climate regulation (Ying and Wei, 2019)—all vital for the health of ecosystem life. Besides, microbes can stimulate rhizodeposition (Bloem et al., 2005) for nutrient acquisition, xenobiotic degradation, metal chelation, trace gas production and consumption, soil acidity, water availability, and hydrophobicity (Fierer, 2017). Regarding the effects of bacteria on agriculture, it has previously been known that some microbes have amazing impacts on plant performance (Jacoby et al., 2017). For example, *B. cereus, B. subtilis* strains, and *Pseudomonas* genus as plant growth-promoting rhizobacteria have been used as biopesticides or biocontrol agents against various plant diseases (Silva et al., 2004; Guo et al., 2019; Basu et al., 2021) and biofertilizers (Shah et al., 2021). The function of improvements in plant health and productivity due to biocontrol bacteria is also mediated by three different ecological mechanisms: antagonism of pests and pathogens, promotion of host nutrition and growth, and stimulation of plant host defenses (induced systematic resistance, ISR) (Choudhary and Johri, 2009; Zheng et al., 2013).

Given the previous reports, microbes are proven to be relatively good promoters for improving plant growth performance and soil quality and alleviating soil erosion on the slope (Normaniza et al., 2018). Since it has been long recognized that unsustainable fertilization practices can contribute to the extensive modification of Earth's biogeochemical cycles through mechanisms such as soil degradation, waterway eutrophication, and greenhouse gas emissions (Jacoby et al., 2017), minimizing the use of chemical fertilizers by using biofertilizers is one of the worthwhile initiatives in sustainable agriculture (Sarkar et al., 2017). In this regard, any factor contributing to microbiome change should be extensively studied. It has been reported that the altitudinal gradient of the sites was a determining factor in the chemical properties of the soil and the distribution of the bacterial communities evaluated (González Mancilla et al., 2018). To date, a variety of studies have investigated the response of the microbial community in bulk soil to various environmental elements, including elevational gradients. It has been shown that elevational gradients strongly affect the microbial diversity and community composition of bulk soils (Meng et al., 2013; Li et al., 2018; Saitta et al., 2018; Guo et al., 2020). It is not always clear whether identified correlations between elevation and diversity represent a direct influence of temperature on microbial communities or whether as confounding factors (Lanzén et al., 2016). The microbiome of soil plays a fundamental role in maintaining the health of soil and plants. While the diversity of microbes is one of the most important factors in the environment, little is known about the effects of elevation on the microbiome and the impact of the affected microbiome on plants. The main goal of this study is to expand our knowledge of what happens to the soil bacterial community along an altitudinal gradient and investigate their possibly different impacts on plant growth.

## Materials and methods

### Site description and soil collection

Soil samples were collected from four sites along an elevation gradient, whose geographic features and physiological parameters are described in Table 1. For each sample, about 2 kg of fresh soil were taken from 0 to 30 cm depth, then immediately brought to the Shahid Beheshti University of Tehran, Iran laboratory, and dried in the dark and at room temperature. Following the drying process, the soil samples passed through a 2-mm sieve and were kept in zip-lock covers. Soil physical properties, including soil texture, pH, EC_e_, and the percentage of clay, silt, and sand, were measured for all soil samples collected from locations 1–4 (Table 1). Synoptic data (2009–2019) from the past 10 years of four sampling sites, including average annual temperature, max temperature, min temperature, average rainfall, average annual speed of the wind, and max wind speed, were obtained from I.R.OF Iran Meteorological Organization (https://www.irimo.ir/eng/index.php).

**Table 1:** Selected geographic and eco-physiological characters of soil samples along the elevational gradient from 2009 to 2019.

**Location description**			**Soil sample no**.			
			**L1**	**L2**	**L3**	**L4**
Geographic characters	Height (meter)		2,365	3,651	4,716	5,179
	Latitude		35°2′24″.77	33°2′22″.20	31°2′0″.78	29°0′17″.19
	Longitude		55°1′35″.20	55°3′12″.91	55°2′18″.09	55°1′39″.96
Physical characters	Percentage of sample weight after sieving		61.04	74.90	55.13	36.04
	pH		7.11	7.76	7.43	7.76
	Ece	ds/m	15.12	1.58	11.40	1.98
	Texture (particle composition)	Sand (%)	44.00	75.00	80.00	68.00
		Silt (%)	47.00	17.00	7.00	17.00
		Clay (%)	9.00	8.00	13.00	15.00
Synoptic characters (10 years Av.)	Temperature (°C)	Lowest value	4.40	7.20	8.40	8.00
		Max value	32.80	33.10	33.40	31.60
		Ann.	18.80	20.50	21.30	19.90
	Total rainfall (mm^3^)	Lowest value	0.50	0.00	0.05	0.40
		Max value	22.40	20.40	11.70	70.10
		Ann.	112.26	99.32	58.71	220.52
	Wind speed (m/s)	Lowest value	1.03	2.75	1.40	2.00
		Max value	2.90	4.30	3.90	3.00
		Ann.	1.90	3.50	2.70	2.60

Av., Average; L1–L4, the sampling areas from north to south of Iran; Lowest value, the least average amount of a parameter; Max value, the maximum average amount of a parameter; Ann., Annual. Sand 2–0.02 mm; silt 0.02–0.002 mm; and clay < 0.002 mm.

### Bacteria isolation procedure

The direct-spreading method was used to isolate soil-borne bacteria, as Chen et al. (2005) described. Therefore, soil samples were processed in a dilution series for inoculating roll tubes and plates. One gram of soil samples was suspended in 2 ml of sterile physiological saline (0.9% w/v NaCl) and vortexed for 1 min. The blend was serially diluted further (usually 10^−1^-10^−7^), and level 100 ul of diluted soil samples were spread on the surface of solidified plates by glass spreaders and incubated in an inverted position at 30°C in the absence of light for 1–3 days. Eleven culture media, including Nutrient Agar (NA), Nutrient Agar plus MnSO_4_ (NA+ MnSO_4_), LB, Moller Hinton Agar (MHA), Acidithiobacillus (APH) medium, Violet Red Bile Lactose (VRB) agar medium, GYM Streptomyces medium, DPM medium, Azospirillum medium, Azotobacter medium, and manure-based medium (MB), were used for bacterial isolation with three replications for each one. To prepare MB medium, dry animal manure and distilled water (1:6 w/v) were mixed and left at room temperature for about 16 h. Then, the resulting mixture was filtered twice, and the remaining extract was centrifuged at 5,000 rcf for 30 min. In the following step, Hoagland salts (10% w/v) were added to the final extract, and the medium was adjusted to pH 5.8 ± 0.02 and autoclaved at 121°C and 1.5 kPa for 20 min. To solidify the medium, bacteriological agar (1.5 w/v) was used as a gelling agent before sterilization.

After bacterial isolation on NA, NA+ MnSO_4_, LB, MHA, APH, VRB, GYM, DPM, and Azospirillum media, the growth of all isolates was evaluated on an MB medium. To investigate isolates biomass in the same condition, we elected MB medium. First, the bacteria were grown in the liquid form of NA, NA + MnSO_4_, LB, MHA, APH, VRB, GYM, DPM, and Azospirillum media at 30°C for 48 h, then 10^3^ cells of each isolate were transferred to 48 well plates containing MB medium, and the plates were incubated at 30°C for 10 h. Then, the growth of bacteria was read at an optical density (OD) of 630 nm 10 h after inoculation. The experiment was performed with three replicates. In the following step, CFU/ml equivalent to each OD was obtained by inoculating the uniform amount of liquid culture of the isolates on the solid form of MB medium at 30°C for 16 h.

### Morphological characterization and biochemical identification of bacterial strains

The soil bacteria were identified by morphologically examining the cell shape, colony shape, colony color, colony size, and biochemical tests. Biochemical characterization was performed by Gram staining, KOH, oxidase, catalase, and urease activity. Gram staining of bacteria was examined after 48 h of incubation on MHA following the method of Coico (2006). The non-staining KOH method was performed to confirm the Gram staining results (Suslow et al., 1982). The catalase test was performed using 0.5 ml of 10% hydrogen peroxide solution and observed to form gas bubbles (Reiner, 2010). The oxidative activity (Schaad et al., 2001) of 68 isolates was studied with biochemical oxidase disks.

### Resistance to abiotic stresses of bacterial isolates

Abiotic stresses resistance of the isolates was measured by screening the resistance to alkaline conditions (pH = 10) (Wiegert et al., 2001), salinity stress (final concentration of 100 mM NaCl) (Gupta et al., 2020), osmotic stress (25% polyethylene glycol Mn6000 PEG) (Vardharajula et al., 2011), and thermal stress (cold stress at 15°C and heat stress at 60°C) (Merkel and Perry, 1977; Graumann et al., 1996; Mukhtar et al., 2020). Muller Hinton media was used as the basic growth culture media for all the experiments, and the incubation period was 15 h, and plates were kept under dark conditions.

### Matrix-assisted laser desorption/ionization-time of flight mass spectrometry sample preparation and analysis

Soil bacterial isolates were subcultured twice on MHA and incubated at 30°C for 24 h before MALDI-TOF MS measurement. Then ~0.1 mg of cell material was directly transferred from a bacterial colony (if possible) or smear of colonies to a MALDI target spot. After drying at laboratory temperature, sample spots were overlaid with 1 μl of matrix solution (10 mg/ml a-cyano-4-hydroxycinnamic acid in 50% acetonitrile and 2.5% trifluoroacetic acid), and each measurement was carried out in triplicate (technical replicates). MS analysis was performed on an Autoflex MALDI-TOF mass spectrometer (Bruker Daltonics, Germany) using Flex Control 3.4 software (Bruker Daltonics, Germany). Calibration was carried out using the Bacterial Test Standard (Bruker Daltonics, Germany). Soil isolates with a valid MALDI-TOF MS score of 2 were undoubtedly assigned to the genus/species level. For bacterial classification, BioTyper 3.1 software (Bruker Daltonics, Germany) equipped with the MBT 6,903 MPS Library (released in April 2016), the MALDI Biotyper Preprocessing Standard Method, and the MALDI Biotyper MSP Identification Standard Method adjusted by the manufacturer (Bruker Daltonics, Germany) were used. A score higher than 2.0 indicated species identification, and a score higher than 1.7 indicated genus identification, whereas any score under 1.7 meant no significant spectrum similarity with any database entry. For the isolated strains, the higher score acquired from the three identification events was used for identification purposes (Avanzi et al., 2017). Only the highest score value of all mass spectra belonging to individual cultures (biological and technical replicates) was recorded (Strejcek et al., 2018).

### Effect of bacterial isolates on plant growth parameters

Maize, canola, and wheat seeds (*Zea mays*. Var Kosha; *Brassica napus* Var Nima; *Triticum aestivum* Var Kalate) were obtained from the Seed and Plant Improvement Institute of Karaj (Karaj, Iran, http://www.spii.ir/en-US/DouranPortal/1/page/Home). Soil-borne isolates grown in MB medium were assayed on maize, canola, and wheat seeds (2 × 10^3^ cell/seed) under greenhouse experiments. The amount of inoculum was calculated by spectrophotometry. In this regard, we have prepared and collected a fresh culture of bacteria. In the next step, collected bacteria have been dissolved in MH and OD of bacteria detected in 630 nm and colony formation unit per milliliter (CFU/ml) calculated and validated by culturing bacteria on a solid medium. The spectrophotometer is an accurate device. In addition, higher concentrations dilute to obtain the desired amount, which gives us a more accurate opportunity. Pot culture experiments were carried out in a greenhouse belonging to the Shahid Beheshti University of Iran. Autoclaved acid-washed sand was applied for planting during the experiments. Seedlings were maintained under a 16/8 h day/night photoperiod at a 25°C temperature for 3 weeks. All treatments in the colonization experiment were carried out under a completely randomized block design and replicated three times. Plant growth parameters include shoot dry biomass (mg), root dry biomass (mg), shoot length (cm), length of root (cm), whole dry weight (mg), whole length (cm), shoot density (mg/cm), root density (mg/cm), and shoot/root weight (mg) were measured under isolate treatment. For mensuration of dry biomass, samples were dried at 60°C for 3 days and then weighted.

### Statistical analysis

The assumption results were presented in the form of a table coded using R software. All the statistical analyses were performed by R software, except for the PCA analysis (version 3.6.2). The significance of the experiment was tested by one-way analysis of variance (ANOVA), and mean separation was performed using Fisher's protected Least Significant Difference (LSD) test at *P* < 0.01 by package Agricolae. ANOVA analyses were applied to all plant–microbe interaction experiments, represented in Supplementary Tables S3–S5, as well as bacterial biomass (**Figure 2A**; Supplementary Table S1). It should be noted that bacterial biomasses were considered based on their growth in the MB medium.

Furthermore, permutational multivariate ANOVA (PERMANOVA) was done by R software (version 4.1.3) for the growth response of the isolates to normal and stresses conditions. Cluster analyses were done based on the Tomida report (Tomida et al., 2002) using CLUSTER (version 3.0) software, and tree images were performed by Java Treeview (version 1.1.6r4). Hierarchical clustering was done based on Euclidian distance and the complete linkage method. PCA analysis was performed by the Clustvist package. The data have been transformed to log_2_ + 1. SVD with the imputation method has been chosen for batching.

## Results

### Sampling locations and eco-physiological characters of soil samples microbiome comparison in different altitude

**Figure 1:**
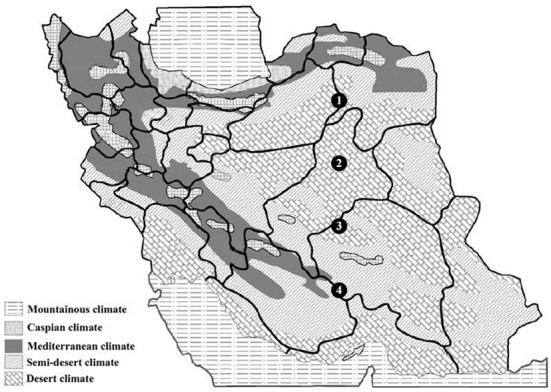
Location of sampling sites on Iran climatic map, from 1 to 4. All samples were taken from semi-arid and arid desert climate regions along a latitudinal gradient. Sampling sites are shown with black circles and for precise location of sampling areas (see Table 1).

**Figure 2:**
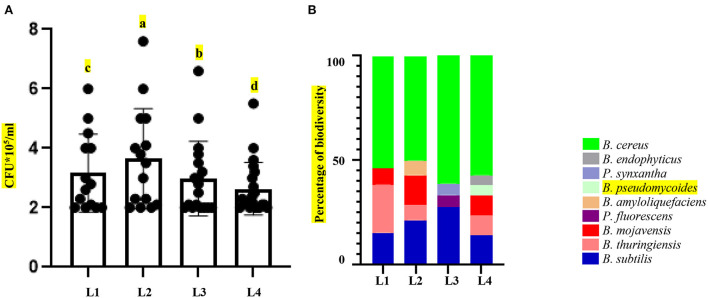
Changes in microbiome in response to altitude gradient. **(A)** Colony counting of microbiome (biomass) of the isolates from soil samples collected in various altitudes. **(B)** Diversity of microbiome isolates collected from various altitudes. Different letters at the top of the graphs indicate statistical differences (*P* value < 0.001) in pairwise comparisons by LSD.

Four sites for the collection of samples were chosen based on comprehensive information on geographical and physical features. Over 10 years of synoptic characteristics of four sampling areas of Iran along an altitudinal gradient (Table 1; Figure 1). The soil samples were taken from a single longitude across four different altitudes; these points were elected on a strip from semi-arid to arid regions from north to south. Accordingly, the altitude difference between sampling areas was from 2,365 to 5,179 m with 1,000-m gaps. Moreover, the triplets' samples were collected from a region with the highest altitude. In the samples with the same general soil texture, marked differences were observed in the sand, silt, and clay percentages (Table 1). Based on soil texture, soil samples belonged to the three soil texture classes. Therefore, the general soil texture in areas first was silt to sandy loam and sandy loam in the rest of the areas. Contrary to minor differences in soil texture, the sampling sites and soil did not differ much in pH levels, temperature, total rainfall, and wind speed. The spectral pH average was 7.11–7.76, which was in the same order. Annual temperatures of the sampling locations were from 18.8° to 21.3°, and as mentioned, total rainfall has not changed more than 100 mm^3^ from each location (Table 1). After sample collection with three replicates, we isolated the bacteria in 11 selective media [11 culture media including Nutrient Agar (NA), Nutrient Agar plus MnSO_4_ (NA+ MnSO_4_), LB, Moller Hinton Agar (MHA), Acidithiobacillus (APH) medium, Violet Red Bile Lactose (VRB) agar medium, GYM Streptomyces medium, DPM medium, Azospirillum medium, Azotobacter medium, and manure-based medium (MB)]. Of 68 soil bacteria, 14 isolates belonged to location L1, 15 to location L2, 18 to location L3, and 21 to location L4 (Supplementary Table S1; Figure 2A). ANOVA analysis of the biomass among four sampling areas (L1–L4) was significant at the *p* < 0.01 level. Interestingly, L2 has the most bacterial biomass while L4 has the least number of bacteria (Figure 2A). MALDI-TOF MS was done on isolates to detect the genus of bacteria. The obtained MALDI-TOF MS profiles were then compared to the reference spectra of the BioTyper database, and their similarity was expressed by a BioTyper Log (score). In total, two different genera of *Pseudomonas* and *Bacillus* were identified. Accordingly, two species belong to the *Pseudomonas* genus: *P. fluorescent* and *P. synxantha. Seven* belong to the *Bacillus* genus: *B. cereus, B. thuringiensis, B. subtilis, B. mojavensis, B. amyloliquefaciens, B. pseudomycoides*, and *B. endophyticus* (Supplementary Table S2). Moreover, the results confirmed by the biochemical tests, as the type and activity of enzymes, such as catalase, oxidase, and KOH degradation produced by the bacteria and gram staining, are important characteristics for identifying the microorganisms (Supplementary Table S2). Diversity analysis shows that *B. cereus* is the dominant and permanent species at any altitude. Second place belongs to *B. subtilis*. Although this bacterium took over 25% of the community, it was not dominant (Figure 2B). Interestingly, the presence of other bacteria in the microbiome could alter *B. subtilis* share of the microbiome in contrast with *B. cereus*. While L3 has the major biomass of bacteria, its diversity suffered, and it contains only *P. fluorescent* and *P. synxantha*. Interestingly, the *Pseudomonas* genus has a unique presence at this altitude. In contrast, L4, which had the lowest biomass, showed the highest diversity in our analysis with the presence of *B. thuringiensis, B. subtilis, B. mojavensis, B. cereus, B. endophyticus*, and *B. pseudomycoides* (Figure 2B).

### Bacteria live in higher altitudes, resist stress, and enhance the growth of plants

**Figure 3:**
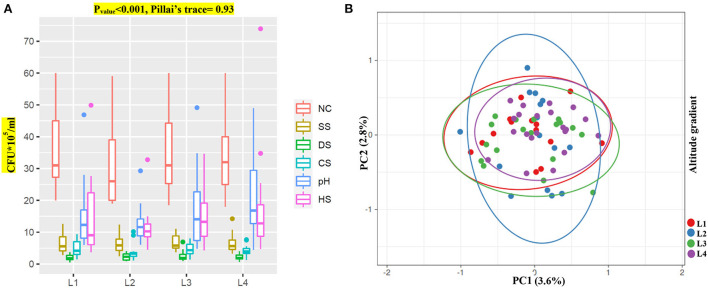
Bacteria growth along an elevational gradient. **(A)** Growth response of bacteria isolates (CFU*10^5^/ml) from different altitudes to NC (normal condition, *F*_value_ = 0.19) and environmental stresses SS (salt stress, *F*_value_ = 0.04), DS (drought stress, *F*_value_ = 0.32), CS (cold stress, *F*_value_ = 0.73), pH (pH stress, *F*_value_ = 1.55) and HS (heat stress, *F*_value_ = 0.60). **(B)** PCA analysis of altitude gradient on bacterial growth.

It is well established that harsh environmental conditions result in evolutionary pressure and eventually the existence of more strong and compatible species. To study the effect of altitude on the microbiome and obtain more suitable bacteria strains with the highest potential, we first tested the stress impact on bacterial isolates. We then tested these bacteria on three valuable plants (maize, canola, and wheat) to see if isolates could beneficially enhance their overall growth. PERMANOVA analysis of the isolate's growth under normal and stress conditions showed significant differences among the sampling locations (Pillai's trace = 0.93, *p* < 0.001). Our data indicate that L3 and L4 bacteria groups have higher resistance to pH and heat stresses and could grow better than other isolates (Figure 3A; Supplementary Table S2). Unfortunately, none of the groups showed any potential for other stresses, such as drought, salt, or low-temperature stresses.

We performed PCA analysis on the stress group to show any differences among the groups and their isolates in each group (Figure 3B). Intriguingly, the PCA test showed convergences among groups with gradual centralization with L4 as a center. As mentioned by an increase in altitude, the centralization of bacteria also increased, which might indicate an evolutionary pressure despite higher diversity in species and strains.

**Figure 4:**
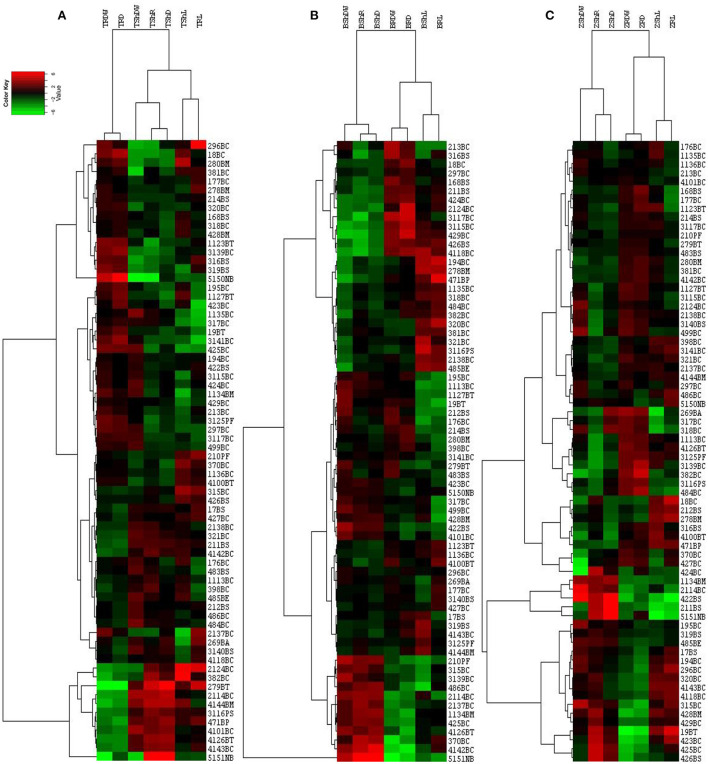
Hierarchical cluster analysis of effect of isolates on wheat **(A)**, canola **(B)**, and maize **(C)** plants, drawn by CLUSTER and Treeview softwares. The horizontal axis shows the plant growth parameters in abbreviation format and the vertical axis shows the bacterial isolates (see Supplementary Table S6). Sh, Shoot; R, Root; L, Length; DW, Dry Weight; D, Density; T, wheat; B, canola; Z, maize or the combination of these abbreviations.

**Figure 5:**
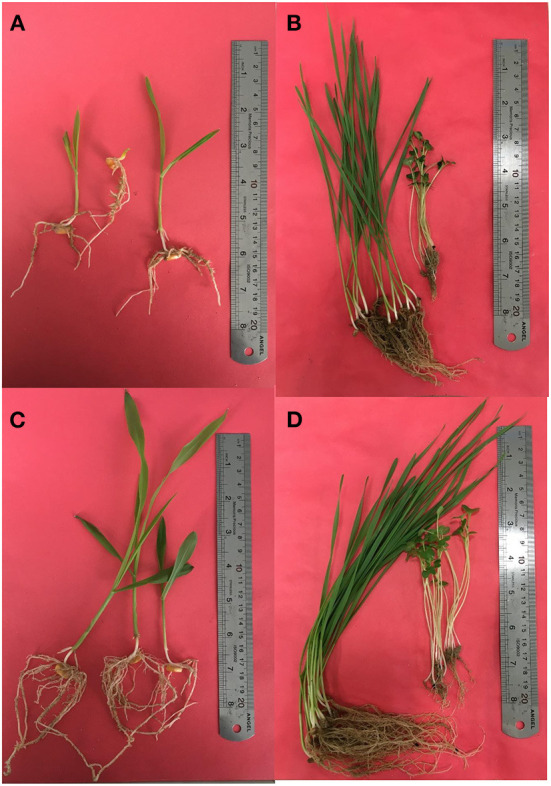
Effects of bacterial isolates on growth of maize, canola, and wheat **(C,D)** in comparison with the uninoculated control condition **(A,B)**.

We also tested the effect of isolates on the growth of plants. The types and sampling elevational gradient of isolates strongly influenced plant growth parameters of maize, canola, and wheat (Supplementary Tables S3–S5; Figures 5C,D) compared to control conditions (Figures 5A,B). As it has been shown, isolates from the L4 and L3 groups have significant effects on root and shoot weight and length in all three plants (Supplementary Table S6; Figures 4A,B, 5). Among these, shoot dry weight, root dry weight, and shoot/root had the greatest difference in terms of influence on *Z. mays* plants (i.e., the most distance and difference on the tree of traits). Based on the arrangement of the isolates in canola traits, clustering shoot/root dry weight, shoot density, root density, and root length had the best impacts on canola plants (Supplementary Table S6; Figure 4). Clustering analysis traits in wheat showed that shoot/root and shoot density had the most significant effects on wheat plants (Supplementary Table S6; Figure 4).

**Figure 6:**
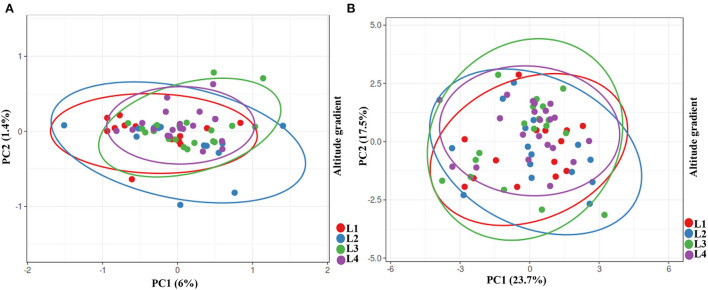
PCA analysis of growth parameters of wheat, maize and canola and comparing isolates in whole. **(A)** Growth parameters of wheat, maize and canola in response to isolates from gradient elevation. **(B)** Batching of isolates based on their effects on plants and their stresses response.

Furthermore, we ran a PCA analysis on the growth parameters of three species as a whole. Once more, we detect a gradual centralization from low to high altitude with L4 as a center, which also supports the PCA microbiome analysis (Figure 6A). This could indicate that each altitude level has a particular microbiome with directional and evolutionary changes. To show the correlation between the stress response data and the effect of isolates on plant growth and to unify the data of isolates, we performed a PCA analysis of isolates with both data sets. Our data, as predicted, show centralized data with L4 as a center of data and lower altitude in the perimeter, which one more time indicates an evolutionary pressure on bacteria despite their diversity (Figure 6B).

## Discussion

The soil microbiome has various roles in the environment. This determining factor is responsible for maintaining soil health and plant growth. Based on the determining role of the soil microbiome, any change that could affect the microbiome is important. In this article, we tried to determine the role of altitude change on soil microbiomes and their mediated impact on plant-microbe changes.

In general, microbial diversity in altitude gradients follows three main mixed patterns: a pattern of decline, a hollow pattern (hump-shaped pattern), and a pattern of increase. There have been a significant number of recent studies addressing how the composition and diversity of soil microorganisms change along elevations (Bryant et al., 2008; Margesin et al., 2009; Djukic et al., 2010; Fierer et al., 2011; Wang et al., 2012; Singh et al., 2014; Yuan et al., 2014; Jin et al., 2018). By contrast, microorganisms (soil bacterial communities at least) do not seem to vary with elevation correspondingly (Bryant et al., 2008; Zhang et al., 2009; Fierer et al., 2011; Singh et al., 2012; Shen et al., 2013, 2014; Yuan et al., 2014). As previously reported and observed, the culturable microflora in rhizosphere/bulk soils along the altitudinal gradient was dominated by microbial species belonging to *Bacillus* and *Pseudomonas* (Rawat et al., 2020). In our study, *B. cereus* was the most abundant bacteria in the microbiome of soils from 2,000 to 5,000 m. Our data suggest that *B. subtilis* could be substituted by other bacteria when altitude changes. We understand that when altitude increases, some bacteria, such as *B. mojavenesis* and *P. fluorescent*, take the place of *B. subtilis*. The *Bacillus* genus produces a wide range of biologically active molecules like lipopeptides that can influence the ecological fitness of the producing strain in terms of root colonization (Dardanelli et al., 2010). Furthermore, *Pseudomonas fluorescens* is a well-known and well-studied species of this genus that has been used as an inoculum to promote plant growth (Li et al., 2017). More importantly, we figured out the dynamics of the microbiome in response to elevation changes. While the microbiome's diversity rises in response to high altitude, its biomass is reduced. Interestingly, high-altitude bacteria flora was resistant to heat and pH changes, while we did not detect any more resistance to other kinds of stress. Hierarchical clustering of growth parameters of maize, canola, and wheat inoculated by high altitude isolates showed enhanced overall growth parameters of plants compared with lower altitudes. These findings have also shown an evolutionary pressure. PCA analysis of plants' growth and bacterial resistance to stresses and PCA from both data sets show that lower altitude isolates show lower similarity. In comparison, higher altitude isolates show similar features despite their higher biodiversity. This could be seen in the centralized pattern of PCA, with a high-altitude group in the center and a low-altitude group on the perimeter of the centralizing pattern. Our data might show that higher altitudes could mildly reduce biomass and, to some extent, increase biodiversity. In addition, our data showed that bacteria living in higher altitudes could be more resistant to heat and pH to some extent.

## Conclusion

Determining altitude changes in the microbiome and their mediated impact on plants could enhance our view of the environmental process of biodiversity and its determinants. Our data back up the role of the higher altitude in an increased amount of biodiversity and lower biomass. Our data also might indicate that bacteria at higher altitudes could somewhat resist heat and pH.

## Data availability statement

The original contributions presented in the study are included in the article/Supplementary materials, further inquiries can be directed to the corresponding author/s.

## Author contributions

MZ carried out the experiment and wrote the manuscript. HA and MS convinced of the presented idea and supervised the project. All authors discussed the results and contributed to the final manuscript.

## Conflict of interest

The authors declare that the research was conducted in the absence of any commercial or financial relationships that could be construed as a potential conflict of interest.

## Publisher's note

All claims expressed in this article are solely those of the authors and do not necessarily represent those of their affiliated organizations, or those of the publisher, the editors and the reviewers. Any product that may be evaluated in this article, or claim that may be made by its manufacturer, is not guaranteed or endorsed by the publisher.
